# Prediabetic changes in gene expression induced by aspartame and monosodium glutamate in *Trans* fat-fed C57Bl/6 J mice

**DOI:** 10.1186/1743-7075-10-44

**Published:** 2013-06-19

**Authors:** Kate S Collison, Nadine J Makhoul, Marya Z Zaidi, Angela Inglis, Bernard L Andres, Rosario Ubungen, Soad Saleh, Futwan A Al-Mohanna

**Affiliations:** 1 Diabetes Research Unit, Department Cell Biology, King Faisal Specialist Hospital & Research Centre, PO BOX 3354, Riyadh 11211, Saudi Arabia; 2 College of Medicine, Al-Faisal University, Riyadh, Saudi Arabia

**Keywords:** Gene expression, Nutrigenomics, Metabolic dysregulation, Adipose, Liver, Aspartame, Monosodium glutamate, Trans-hydrogenated fat

## Abstract

**Background:**

The human diet has altered markedly during the past four decades, with the introduction of *Trans* hydrogenated fat, which extended the shelf-life of dietary oils and promoted a dramatic increase in elaidic acid (*Trans*-18.1) consumption. Food additives such as monosodium glutamate (MSG) and aspartame (ASP) were introduced to increase food palatability and reduce caloric intake. Nutrigenomics studies in small-animal models are an established platform for analyzing the interactions between various macro- and micronutrients. We therefore investigated the effects of changes in hepatic and adipose tissue gene expression induced by the food additives ASP, MSG or a combination of both additives in C57Bl/6 J mice fed a *Trans* fat-enriched diet.

**Methods:**

Hepatic and adipose tissue gene expression profiles, together with body characteristics, glucose parameters, serum hormone and lipid profiles were examined in C57Bl/6 J mice consuming one of the following four dietary regimens, commencing *in utero* via the mother’s diet: [A] *Trans* fat (TFA) diet; [B] MSG + TFA diet; [C] ASP + TFA diet; [D] ASP + MSG + TFA diet.

**Results:**

Whilst dietary MSG significantly increased hepatic triglyceride and serum leptin levels in TFA-fed mice, the combination of ASP + MSG promoted the highest increase in visceral adipose tissue deposition, serum free fatty acids, fasting blood glucose, HOMA-IR, total cholesterol and TNFα levels. Microarray analysis of significant differentially expressed genes (DEGs) showed a reduction in hepatic and adipose tissue PPARGC1a expression concomitant with changes in PPARGC1a-related functional networks including PPARα, δ and γ. We identified 73 DEGs common to both adipose and liver which were upregulated by ASP + MSG in *Trans* fat-fed mice; and an additional 51 common DEGs which were downregulated.

**Conclusion:**

The combination of ASP and MSG may significantly alter adiposity, glucose homeostasis, hepatic and adipose tissue gene expression in TFA-fed C57Bl/6 J mice.

## Introduction

Obesity and nonalcoholic fatty liver disease (NAFLD) are comorbid conditions which are increasingly prevalent throughout the world [1]. Several dietary factors have been implicated as causative, including excessive sucrose, fructose and fat [2-4]. The human diet has changed greatly during the past four decades, with the introduction of partially-hydrogenated vegetable oils (*Trans* fats), which began replacing natural fats and oils in processed foods during the 1960s. *Trans* fat (TFA) has no nutritional value, and has been associated with major health deficits [5]. In 1994, it was estimated that TFA consumption could be linked to the approximately 20,000 fatalities from heart disease annually in the United States alone [6]. Increased abdominal adiposity has been shown to result from TFA-feeding in monkeys [7]; and increased visceral fat and hepatic lipid accumulation together with impaired insulin sensitivity were noted in rats consuming a low fat diet supplemented with 4.6% elaidic acid (*Trans*-18.1), which is the predominant lipid found in hydrogenated vegetable oils [8]. Furthermore, we have previously investigated the changes in hepatic and adipose tissue gene expression which accompany the increased adiposity and hepatic steatosis induced by TFA supplementation, and noted significant dysregulation in several key transcription factors controlling energy metabolism [9].

In addition to extending the shelf life of foods and reducing production costs, processed foods can be rendered more palatable by the use of food additives to preserve flavor and enhance taste [10]. Two of the most commonly consumed food additives are monosodium glutamate (MSG) which enhances flavor when combined with another savory odor [11]; and the low-calorie artificial sweetener aspartame [12], used to replace dietary sugar and fructose. However in rodents, neonatal injections of high doses of MSG promotes obesity and growth hormone defects together with hyperinsulinemia and elevated corticosteroid levels in adulthood [13-16]. This hypothalamic model of obesity may also be induced in the offspring of pregnant dams orally ingesting MSG [17-19]; and studies with radiolabeled ^3^H-glutamate have shown that glutamate given orally to pregnant mice can subsequently be detected in the maternal and fetal brains, livers and kidneys [20]. The mechanism behind the neuroendocrine disturbance caused by MSG is believed to involve glutamate-induced degeneration of those areas of the immature neonatal brain which are insufficiently protected by a mature blood–brain barrier, including regions which regulate growth and energy metabolism [13-19].

MSG has previously been demonstrated to cause hepatic microsteatosis and a NASH-type phenotype in rodents exposed to MSG neonatally [21]. We have previously shown that the combination of TFA and MSG can further modulate hepatic and adipose tissue gene expression, by increasing the transcription of genes involved in lipid mobilization and storage, together with an attenuation of the expression of several lipid catabolizing genes [9]. In both tissues, transcription factor Srebp1c was increased by TFA consumption; whereas in the adipose tissue, expression of the key transcription factor Peroxisome proliferator-activated receptor-gamma coactivator-1alpha (Ppargc1a) was reduced by 50% in TFA-fed mice, and reduced further to 25% by the combination of TFA and MSG. Ppargc1a is a potent transcriptional activator of glucose and lipid metabolism linking nutritional and hormonal signals and energy metabolism; and Ppargc1a-null mice develope systemic dyslipidemia and hepatic steatosis [22].

The low-calorie dipeptide artificial sweetener aspartame (ASP) is rapidly metabolized upon ingestion into its metabolic components phenylalanine, aspartate and methanol, in the ratio of 50:40:10 w/w/w [23]. Recently, chronic consumption of aspartame by mature rodents has been shown to cause hepatocellular injury and a reduction in hepatic antioxidant defense capacity [24]. Additionally, we have utilized a rodent model in order to investigate the interactive effects of chronic exposure to ASP and MSG, either alone or in combination [25]. We demonstrated that C57Bl/6 J mice exposed to ASP both *in utero* via the mother’s diet, and subsequently throughout the first five months of life, developed hyperglycemia and reduced insulin tolerance; and that the combination of ASP and MSG appeared to interact in further modulating insulin sensitivity in young C57Bl/6 J mice. Throughout the study, animal exposed to ASP and MSG were maintained on a Standard Chow diet, which has a relatively low fat content, (typically of between 5-10%), however this may not accurately reflect current dietary trends in the human population [26]. We were therefore interested to know how consumption of ASP and MSG might affect the physiology and gene expression of animals consuming a TFA -enriched diet, since in processed foods and restaurant fare, TFA, MSG and aspartame may frequently be consumed together in the same meal. Since TFA [7-9,27], ASP [24] and MSG [9,21] have all been implicated in hepatic dysfunction, our aim was to examine the development of hepatic steatosis, adiposity and changes in hepatic and adipose tissue gene expression in response to ASP and MSG, or a combination of both, in animals maintained on a TFA -enriched diet.

Because exposure to nutritional and environmental challenges during critical periods of early development can markedly effect metabolism in later life [28], and since differentiation of the rodent neuroendocrine system regulating energy homeostasis begins during gestation and continues for a significant period of time after birth [29], our study animals were exposed to these dietary manipulations *in utero* via the mother’s diet and throughout the first five months of life, using an experimental design similar to our previous NAFLD studies [9,30]. Concentrations of ASP and MSG used throughout this study were comparable with the Acceptable Daily Intake for both substances [31,32], which we believe makes our experimental paradigm an appropriate model for examining the interaction between commonly-consumed food additives. To our knowledge this is the first article to address the effect of these additives on hepatic and adipose tissue gene expression in a rodent nutrigenomics model.

## Materials and methods

### Animals and diets

Our study animals were bred from C57Bl/6 J mice obtained from The Jackson Laboratory (Maine, USA). Female breeders were housed and fed a standard chow diet until 6 weeks of age whereupon they were placed on one of 4 different dietary regimens for an adjustment period of 3 weeks prior to mating as described previously [9,30]. The four diet regimens used in this study were: [1]**TFA control diet**: consisting of 20% (w/w) Partially Hydrogenated Vegetable Shortening (Test Diet #5C4M containing 8.68% w/w *Trans* fatty acids; Test Diet Purina, USA), with *ad lib* drinking water [2]. **MSG + TFA diet**: TFA diet with *ad lib* drinking water containing 0.75 g/L of L -Glutamic acid monosodium salt hydrate (MSG catalog G1626 Sigma Aldrich) [3]. **ASP + TFA diet**: TFA diet with *ad lib* drinking water containing 0.25 g/L Asp-Phe methyl ester (aspartame, ASP, catalog A5139 Sigma Aldrich) [4]. **ASP + MSG + TFA diet:** TFA diet with *ad lib* drinking water containing 0.25 g/L aspartame and 0.75 g/L monosodium glutamate (see Additional file 1 for diet composition). Following mating, the 4 groups of breeder dams were maintained on their respective diets throughout the gestation, birth and nursing period. The offspring used in the following experiments were weaned onto the same maternal dietary regimen at 4 weeks of age and maintained on their respective diets for the duration of the study. Male offspring (n = 15 per diet group) were housed 3 to a cage in an identical manner as described above. Average body weight was assessed at 6 & 17 weeks of age. Percentage weight change between these two time-points was calculated as follows:

$$\begin{array}{ll} \%\;\mathrm{weight}\;\mathrm{change}\;= & \;(\mathrm{weight}\;\mathrm{at}\;17\;\mathrm{weeks}-\\ & \;\mathrm{weight}\;\mathrm{at}\;6\;\mathrm{weeks})/\mathrm{weight}{\;}^{*}100 \end{array}$$

mean ASP and MSG consumption were calculated from the amount of aspartame-water and MSG-water consumed, and expressed in mg per Kg body weight. At the conclusion of the study (20 weeks of age), overnight-fasted subjects were euthanized with a mixture of xylazine and ketamine, and the blood was collected for analysis of serum components. Concomitantly, the liver and the visceral fat (epididymal fat pads, together with adipose tissue associated with the reproductive organs and omental-mesenteric fat associated with the digestive organs) was carefully excised, rinsed in PBS buffer, blotted dry and weighed to the nearest 0.01 g. Tissues were either snap-frozen in liquid nitrogen for RNA isolation and hepatic lipid quantitation, or immediately placed in 5× volume of formalin for histological analysis. The breeding and care of the animals were in accordance with the protocols approved by the Animal Care and Use Committee of the King Faisal Specialist Hospital & Research Centre.

### Measurement of fasting serum glucose, hormone, and lipid profile

Overnight fasting blood glucose was measured from the tail vein of the 6-week and 17-week old experimental subjects using the Ascensia Contour glucometer (Bayer HealthCare, IN, USA). Additionally, total cholesterol (T-CHOL), and HDL-C concentrations were also measured in overnight fasted 17-week old mice using the Reflovet Plus instrument (Roche, F. Hoffmann-La Roche Ltd, Basel, Switzerland) as described in our previous studies [9,30]. A panel of murine hormones and cytokines relating to the metabolic syndrome (amylin, C-peptide, gastric inhibitory peptide GIP, insulin, leptin, MCP-1, resistin, and TNFα) were simultaneously analyzed using the Multiplex bead-based Mouse metabolic assay catalog# MMHMAG-44 K (Millipore, Billerica, MA, USA), according to manufacturer’s instructions. Samples and standards were processed using the Luminex 100/200 system with related xPONENT^®^ software (version 3.1; Luminex Corporation, Austin, TX, USA). A five-parameter logistic regression model with weighting was used to create standards curves (pg/mL) and calculate the mean of sample concentration from each triplicate. Homeostatic Model Assessment Index (HOMA-IR) values, a measure of insulin resistance, were calculated according to the established formula: (fasting serum glucose mM) * (fasting serum insulin μIU/ml)/22.5 [33].

### Liver and adipose tissue histology, and hepatic triglyceride quantitation

Formalin-fixed, paraffin-embedded liver and visceral cavity adipose tissues from each of the experimental subjects were processed, and 4 μm-thick serial sections were cut and stained with hematoxylin and eosin (H&E: liver) or trichrome (adipose tissue) according to standard laboratory procedures. After mounting with glycerol gelatin, images were captured using Axio Vision Rel4.5 software (Carl Zeiss). Levels of mouse liver triglyceride (Tg) were quantified using the Triglyceride Determination Kit TRO100 with appropriate triglyceride standards (Sigma Aldrich, MO, USA). Frozen liver samples from 20-week old mice were first powdered under liquid nitrogen and 120 mg of the frozen liver powder was weighed into 2 ml chloroform: methanol mix (2:1 v/v) and incubated for 2 hours at room temperature with continuous shaking. Following the addition of 0.2 vols H_2_O, vortexing and centrifuging at 2500 g, the lower phase chloroform containing the lipids was collected and dried under vacuum in a rotary evaporator for 5–6 hr. The dried pellets were resuspended in the reaction buffer provided in the Kit. Results were expressed as mean Tg (mg /g tissue) ± SEM, n = 15 per diet group.

### RNA isolation

Animals (n = 15 per diet group) were euthanized at 20 weeks of age by xylazine/ketamine intramuscular injection, and the liver and adipose tissues were rapidly removed and rinsed in PBS buffer as described above. After weighing, the tissues were snap-frozen for RNA extraction as described previously [9,30]. Total RNA was prepared using Qiagen RNeasy Kit (Qiagen, CA, USA) according to the manufacturer’s instructions and stored at −80°C. RNA integrity was measured using a 2100 Bioanalyzer instrument and an RNA 6000 Nano LabChip assay (Agilent Technologies, CA, USA). RNA concentrations were determined by absorption at 260-nm wavelength with an ND-1000 spectrometer (Nanodrop Technologies, DE, USA).

### Gene expression analysis

**Figure 1:**
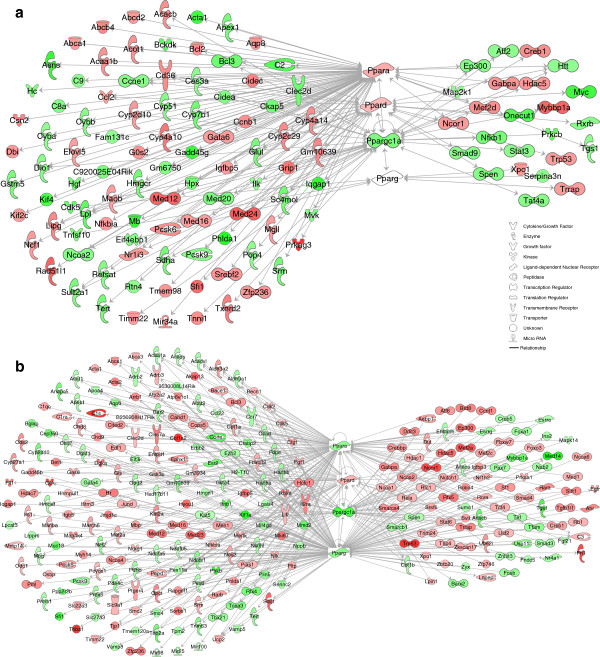
**Functional relationship networks of PPAR and PPAR-interacting DEGs responsive to ASP + MSG.** IPA-generated networks depict PPAR-interacting DEGs in **(a)** Liver &**(b)** Adipose (P < 0.01, stringency ≥ ± 1.4-fold change in expression).

Gene expression in these samples was analyzed using 12 GeneChip (R) Mouse Gene 1.0 ST arrays representing 28,853 genes. We used 3 chips per diet group, and applied pooled RNA from 5 mice per chip (see Figure 1 for design of the microarray experiments). Targets were prepared and microarrays were processed as described in the Affymetrix GeneChip Whole Transcript Expression Analysis manual using the Ambion WT expression kit and Affymetrix WT Terminal Labeling Kit as per manufacturers’ instructions. Briefly, approximately 250 ng of total RNA was used to synthesize double-stranded DNA with random hexamers tagged with a T7 promoter sequence. The cDNA was used as a template for *in vitro* transcription. In the second cycle cDNA synthesis, random primers were used in reverse transcription to convert the cRNA into single-stranded DNA, which was fragmented, labeled, and hybridized to the array for 17 hours, then washed and stained using the Fluidics 450 station (Affymetrix, Santa Clara, CA). Arrays were scanned using the Affymetrix 3000 7G scanner and GeneChip Operating Software version 1.4 to produce.CEL intensity files. This software also provided summary reports by which array QA metrics were evaluated including average background, average signal, and 3′/5′ expression ratios for spike-in controls, β-actin, and GAPDH. Microarray data was deposited at the MIAME compliant NCBI gene expression hybridization array data repository (GEO: http://ncbi.nlm.nih.gov/geo) under accession #GSE38444 and GSE38445 (expression data from liver and adipose tissue respectively).

### Quantitative PCR

Confirmation of microarray results was performed by quantitative PCR (qPCR) as previously described [30]. Total RNA (2 μg) was reverse transcribed using SuperScript II reverse transcriptase (Invitrogen, CA, USA). Subsequently, qPCR reactions were performed in triplicate using SYBR-Green 1 master mix (Roche Diagnostics IN, USA) and 10 ng cDNA as template. No template and no reverse transcriptase controls were included and products were analyzed by gel electrophoresis. Real time qPCR values for each target gene were calculated as a ratio of target gene expression level to GAPDH expression level in the same specimen. Statistical significance was assessed using a two-tailed t test assuming unequal variance of the biological replicates. Intron-spanning gene specific primers were designed using Primer 3 software and sequences are provided in Additional file 2: Table S2. DEGs that differed significantly (P < 0.01) in their regulation between the diet groups’ microarray analysis were selected, based on their biological relevance, and validated with the same samples by qPCR analysis. Ratios of expressions between the diet comparisons were calculated from the microarray data set and Pearson correlation analysis between the qPCR and microarray data were calculated.

### Data analysis

Morphological and biochemical data were analyzed using one-way analysis of variance (ANOVA) followed by Tukey’s test. Differences were considered significant at P < 0.05. For identification of differentially expressed genes (hereafter termed DEGs), microarray analysis was performed using the Partek Genomic suite software version 6.3 (Partek, MO, USA) as previously described [9,30]. Probe set data were summarized and background adjusted using the GC-Robust Multi-Array (GCRMA) algorithm [34], as implemented in the microarray analysis software package. The GC-RMA algorithm is based on a linear additive model, and assumes that *Y*_*gij*_ = *O*_*gij*_ + *N*_*gij*_ + *S*_*gij*_, where *Y*_*gij*_ is the perfect match probe intensity value for probe *j* in probeset *g* on array *i*. *O*_*gij*_ is the corresponding “optical noise” due to laser scanning errors, *N*_*gij*_ is the corresponding “non-specific binding noise,” and *S*_*gij*_ is a quantity proportional to the actual abundance of target transcript in a sample, which allows for estimation of the “true” expression value. This method is used to reduce discrepancies in hybridization patterns from each of the three chips per diet group that might result from variables in target amplification, hybridization conditions, staining or probe array lot. All data were normalized using non-linear transformation termed Quantile Normalization to improve the linearity, normality & homoscedasticity of the data, which was further filtered to remove noise and extreme expression values. Additionally, all probesets without unique Entrez gene identifiers were removed from the analysis. We used a one-way ANOVA to test for diet interactions without correction for multiple testing. A significance value of 0.05 indicated that the gene was differentially regulated by diet. See Figure 1 for the design and analysis of the microarray experiments. In order to identify genes regulated in response to dietary manipulation, we used the False Discovery Rate (FDR) method [35] in which p-values were adjusted simultaneously across multiple subgroup comparisons. Contrasts were included in the model based on the comparison of interest. All further sub-lists were created using genes that passed the FDR adjusted ANOVA p-value as well as fold change criteria. A significance value of 0.05 indicated that the gene was differentially regulated by diet with a stringency of 1.4-fold change in expression values. For identification of diet-induced differentially expressed genes we considered the comparison of three conditions (TFA versus ASP + TFA; TFA versus MSG + TFA; and TFA versus ASP + MSG + TFA). All resultant genes and expressed sequence tags (ESTs) meeting the criteria for ASP- and MSG-induced differential expression were classified as relatively upregulated or downregulated compared to TFA. Genes fulfilling these criteria were ascribed genome-wide significance using the Database for Annotation, Visualization, and Integrated Discovery (DAVID) annotation [36]. Ingenuity Pathways Analysis (IPA: http://www.ingenuity.com) was used for detection of gene ontology, canonical pathways analysis, with significant transcripts in diet groups compared to control. Networks of biologically related genes were also created using IPA. Heat maps of the gene/ESTs expression data were generated using Matlab (version 7.6, The MathWorks, Inc. USA) and Partek.

## Results

### A combination of aspartame and MSG in a *Trans* fat diet promotes hyperglycemia, and increases adiposity and hepatic steatosis

The experimental design is outlined in Additional file 3. There were no significant differences in food and water intake between the 4 different diet groups (data not shown). Mean TFA consumption was 0.02 g/Kg body weight. Mean aspartame (ASP) and monosodium glutamate (MSG) consumption in the drinking water was 43.54 ± 1.42 mg/Kg body weight and 130.03 ± 5.61 mg/Kg body weight respectively. Histologically, livers presented with indications of micro- and macrosteatosis, with the ASP + MSG diet apparently resulting in the highest degree of overall steatosis compared to the other diet groups (see Additional file 4). Trichrome staining of adipose tissue taken from visceral depots showed a non-significant increase in the apparent size of adipose tissue cells.

Whilst there were no apparent differences in the mean body weight or weight of liver tissue between all 4 diet groups, the weight of visceral adipose tissue in the ASP + MSG + TFA diet group was significantly greater than all three other diet groups, suggesting the development of obesity (Table 1, P = 0.001). Additionally, levels of plasma free fatty acids (FFA) were significantly elevated in both MSG-containing diet groups (MSG + TFA and ASP + MSG + TFA). These two diet groups also had the highest levels of hepatic triglyceride (TG) content indicative of hepatic steatosis (P ≤ 0.001).

**Table 1:** Body characteristics and biochemical, lipid and hormone profile of experimental subjects

	**TFA**	**MSG + TFA**	**ASP + TFA**	**ASP + MSG + TFA**	**Sig.**
Body Weight (g)					
6-week	20.07 ± 0.43	21.29 ± 0.28	20.94 ± 0.26	21.03 ± 0.32	0.06
17-week	31.58 ± 0.77	32.99 ± 0.59	31.37 ± 0.58	32.36 ± 0.87	0.31
% Weight Change	57.58 ± 2.85	54.91 ± 1.59	50.19 ± 3.44	54.78 ± 2.38	0.27
Adipose Weight (g)	1.08^**a**^ ± 0.13	1.36^**ab**^ ± 0.09	0.98^**a**^ ± 0.16	1.67^**b**^ ± 0.14	0.001
Liver Weight (g)	1.25 ± 0.06	1.23 ± 0.05	1.30 ± 0.03	1.33 ± 0.06	0.49
Liver Triglyceride (mg/ml)	8.12^**a**^ ± 0.65	12.14^**b**^ ± 1.00	9.90^**ab**^ ± 1.16	13.33^**b**^ ± 0.84	0.001
T_CHOL (mmol/dL)	121.60^**a**^ ± 1.34	121.67^**a**^ ± 3.25	127.0^**ab**^ ± 1.62	130.13^**b**^ ± 1.54	0.01
HDL (mmol/dL)	87.43 ± 4.08	89.39 ± 4.61	96.75 ± 1.22	98.09 ± 2.91	0.08
Free Fatty Acids (μM)	156.99^**ab**^ ± 4.59	177.36^**bc**^ ± 7.73	146.2^**a**^ ± 5.30	190.03^**c**^ ± 5.36	<0.0001
Fasting Glucose (mg/dL)					
6-week	45.00^**a**^ ± 2.10	52.03^**ab**^ ± 3.41	62.50^**b**^ ± 3.09	92.50^**c**^ ± 3.87	<0.0001
17-week	98.53^**ac**^ ± 8.05	117.43^**ab**^ ± 10.21	81.97^**c**^ ± 6.53	132.90^**b**^ ± 8.23	<0.001
Insulin (pg/ml)	525.5 ± 55.6	552.51 ± 56.57	539.73 ± 43.84	606.46 ± 54.50	0.75
HOMA-IR	3.65^**a**^ ± 0.38	4.67^**ab**^ ± 0.27	3.06^**a**^ ± 0.31	5.86^**b**^ ± 0.70	<0.001
C-Peptide (pg/ml)	1761.88^**a**^ ± 200.3	1708.5^**a**^ ± 123.17	1078.42^**b**^ ± 86.51	1994.65^**a**^ ± 150.65	<0.001
Amylin (pg/ml)	58.38 ± 5.39	58.82 ± 5.55	64.74 ± 4.24	59.75 ± 4.82	0.81
GIP (pg/ml)	129.13^**ab**^ ± 17.9	95.46^**b**^ ± 7.37	143.68^**c**^ ± 11.91	113.75^**ac**^ ± 8.69	0.04
Leptin (pg/ml)	5096.68^**a**^ ± 662.7	9073.34^**b**^ ± 1319.8	5460.9^**a**^ ± 979.17	10917.55^**b**^ ± 716.01	<0.001
MCP-1 (pg/ml)	69.06^**a**^ ± 7.26	55.09^**a**^ ± 6.03	69.05^**a**^ ± 7.96	39.58^**b**^ ± 4.53	0.01
Resistin (ng/ml)	25.24 ± 1.64	23.12 ± 1.33	21.64 ± 1.24	25.33 ± 1.67	0.26
TNF-α (pg/ml)	11.35^**a**^ ± 1.35	7.77^**a**^ ± 1.97	9.59^**a**^ ± 1.40	30.56^**b**^ ± 11.44	0.03

*HOMA*-IR Homeostasis model of assessment of insulin resistance, *GIP* Gastric inhibitory polypeptide, *MCP*-1 Monocyte chemotactic peptide-1. Data are presented as Means ± SEM (n = 15 per diet group). Significant differences in Means are indicated using different letters *abc* for p-value of <0.05.

Fasting blood glucose levels were highest in the ASP + MSG + TFA diet group at both 6 and 17 weeks of age (Table 1, P < 0.001). At 17 weeks of age, fasting blood glucose levels were markedly higher than the levels at 6 weeks; and the combination of ASP + MSG in the TFA -fed mice elevated fasting glucose to pre-diabetic levels [37]. Analysis of hormones and cytokines related to the development of obesity and hepatic steatosis showed that both MSG-containing diet groups had significantly higher levels of serum leptin and TNFα (MSG + TFA and ASP + MSG + TFA diet mice, Table 1, P <0.05). Fasting serum insulin levels were increased by 15% in ASP + MSG + TFA diet mice although this observation failed to reach statistical significance. However, the HOMA-IR index, an indicator of insulin resistance, was significantly increased by 56% in ASP + MSG + TFA diet mice (P < 0.001). Levels of serum amylin and resistin were not significantly affected by the food additive diets. In summary, the addition of MSG to the TFA diet raised hepatic triglyceride and serum leptin levels. The combination of ASP + MSG promoted visceral adiposity, and elevated hepatic triglyceride levels, serum FFA, fasting blood glucose, TNFα and HOMA-IR index in *Trans*-fat fed mice.

We next used Pearson correlation analysis In order to further understand the mechanism behind the increased hepatic triglycerides and visceral fat observed in the ASP and MSG-fed mice. Significant correlations between levels of hepatic triglyceride content and adipose tissue weight, FFA, serum cholesterol and leptin were apparent, suggesting the possibility that the mechanism behind the lipid accumulation in the liver could be via increased adipose tissue mass and flux of FFA to the liver. HOMA-IR, a strong indicator of the development of insulin resistance, correlated with body and adipose tissue weight, fasting glucose, insulin, C-peptide, leptin and resistin (Table 2, P < 0.05).

**Table 2:** Correlations of HOMA-IR and hepatic triglyceride (TG) levels with markers of weight gain, adiposity and dyslipidemia

	**Liver TG (mg/ml)**	**HOMA-IR**
Body weight (g)		
6-week	.031	.338^*^
17-week	.319^*^	.575^**^
Weight gain (%)	.355^**^	.387^**^
Adipose weight (g)	.565^**^	.551^**^
Liver weight (g)	.358^**^	.174
Liver TG (mg/ml)	1	.263
T_CHOL (mmol/dL)	.439^**^	-.027
Free fatty acids (μM)	.361^*^	.119
Fasting glucose (mg/dL)		
6-week	.268^*^	.436^**^
17-week	.428^**^	.693^**^
Insulin (pg/ml)	-.063	.742^**^
HOMA-IR	.260	1
C-peptide (pg/ml)	.235	.620^**^
Leptin (pg/ml)	.555^**^	.519^**^
MCP-1 (pg/ml)	-.376^*^	-.207
Resistin (ng/ml)	-.044	.403^**^

Significant correlations are indicated in bold by ** at the 0.01 level and * at the 0.05 level (2-tailed). *TG* triglyceride.

Significant correlations are indicated in bold by ** at the 0.01 level and * at the 0.05 level (2-tailed). *TG* triglyceride.

### Aspartame and MSG modify hepatic and adipose tissue gene expression

Microarray analysis of hepatic and adipose tissue gene expression was used in order to further examine the mechanism behind the diet-induced changes in adiposity and glucose homeostasis. We used Affymetrix Mouse Gene 1.0 ST expression arrays to determine differences in global gene/Expressed Sequence Tags (ESTs), identifying10,117 hepatic gene transcripts and almost 3 times that number (28,101) of adipose tissue transcripts, with a significant p-value of 0.05 for diet (see Additional file 3). In order to identify the impact of consumption of ASP, MSG or a combination of both on gene expression in *Trans* fat-fed mice, we next identified subsets of ASP-induced and MSG-induced DEGs using a false discovery rate as described by Benjamini and Hochberg, with a significance set at 0.05 and a stringency of ± 1.4-fold. We identified 8327 liver transcripts which were differentially expressed by either ASP, MSG or a combination of the two compared to the TFA-fed control, and a corresponding 11,600 adipose tissue transcripts. These DEGs were further bifurcated into either down- or up-regulated in response to the food additives (see Additional file 5). We identified subsets of 1715 DEGs upregulated by ASP in the liver and 2599 DEGs upregulated by ASP in the adipose tissue. In both tissues, MSG upregulated the lowest number of DEGs: 1017 in the liver and 1249 in the adipose tissue. Interestingly, the combination of ASP + MSG upregulated the highest number of DEGs in the adipose tissue (2691) compared to approximately half as many in the liver (1148). Broadly speaking, the number of ASP- and MSG-induced downregulated DEGs was similar to the number upregulated, the exception being in the adipose tissue, where MSG down-regulated approximately half the number of upregulated DEGs. In summary, in terms of global gene activation, the adipose tissue appears to be more responsive to dietary manipulation compared to the liver, and the combination of ASP + MSG induced the highest number of DEGs compared to either food additive alone.

### Functional gene ontology analysis of food additive-responsive DEGs

In order to further classify the nature of the food additive-responsive gene activation in TFA -fed mice, we examined the functional gene ontologies of the diet-responsive DEGs in both liver and adipose tissue. Additional file 6 summarizes the main significant ontologies of up- and down-regulated DEGs induced by ASP, MSG or a combination of the two, in the livers and adipose tissue of TFA -fed mice. MSG induced hepatic gene expression related to blood vessel morphology, transportation of molecules, and uptake of lipids. Conversely, genes related to RNA expression, proliferation, mitosis and xcell death were down-regulated. In adipose tissue, MSG induced genes with ontologies related to vasculogenesis, proliferation and quantity of mononuclear leukocytes, whereas genes relating to glucose metabolism disorders and Diabetes Mellitus were down-regulated (p < 0.05).

A different pattern of gene expression ontologies was seen in ASP-treated mouse tissues, with the significant upregulation of genes involved in the organization of the cytoplasm, morphology of blood vessels and angiogenesis, and the down-regulation of genes with ontologies relating to hepatic protein metabolism, organization of peroxisomes and expression of RNA (see Additional file 6). ASP-induced genes relating to cell proliferation and the development of blood vessels were upregulated in the adipose tissue of TFA -fed mice; whereas down-regulated ontologies included adipogenesis and the proliferation of immune cells. Common gene ontologies significantly induced by the combination of ASP + MSG included morphology of vessels, vasculogenesis and fatty acid metabolism, whereas hepatic genes relating to hepatic expression of RNA and cell cycle progression was downregulated in all 3 cases. In adipose tissue, similarities in the ontologies of significant DEGs upregulated by both food additives included vasculogenesis, angiogenesis and response to macrophages, whereas down-regulated DEGs related to proliferation of leukocytes and the metabolism of glucose.

### Generation of networks of diabetogenic peroxisome proliferator-activated receptor (PPAR) genes

Since the combination of MSG and aspartame promoted the largest changes in adiposity, blood glucose and lipid dysregulation in TFA -fed mice, we next generated networks of differentially expressed genes which were dysregulated in response to both ASP and MSG feeding. We were particularly interested in transcripts associated with the dysregulation of PPAR genes, since it is known that PPAR transcriptional activity is affected by diet and adiposity, and particularly dysregulated by diabetes [37]. Figure 1a shows networks of significant hepatic PPAR DEGs up-regulated (red) or down-regulated (green) by the combination of ASP + MSG in the livers of TFA -fed mice. Key “Master switch” transcription factor Ppargc1a expression was reduced by 1.7-fold, conversely levels of hepatic Pparα and Pparδ were modestly increased in this experimental model. Lipogenic cholesterol homeostasis transcription factor Srebf2 was upregulated 1.6-fold, together with a similar increase in cyclic AMP responsive element binding protein Creb1 (P < 0.05). Additionally, expression of Acetyl-CoA carboxylase ß (Acacb, Acc2), an important diet-regulated enzyme involved in lipogenesis was increased 1.9-fold together with a 1.8-fold increase in carnitine palmitoyltransferase 1c (Cpt1c), one of the first enzymes in the mitochondrial ß-oxidation of long chain fatty acids. Another feature of the food-additive induced gene expression was the upregulation of PPAR target genes Acetyl CoA thioesterase (Acot1), CD36 (1.7-fold) and endothelial lipase (Lipg: 1.7-fold) which are involved in lipid hydrolysis. PPARα-related genes involved in cholesterol and phospholipid homeostasis such as the ATP-binding cassette transporters Abca1, Abcb4 and Abcd2 were upregulated approximately 1.5-fold, additionally we found a 1.7-fold upregulation of the PPARα marker genes Cyp4a10 and Cyp4a14 and acetyl-Coenzyme A acyltransferase (Acaa1b); all previously identified as being upregulated in models of diabetes. Several genes involved in cellular stress induction and protection were deregulated by ASP and MSG in TFA-fed mice, including an increase in Trp53 (p53: 1.42-fold), Car (Nr1i3; 1.43-fold), Mef2d (1.6-fold) and Hdac5 (1.5-fold); together with a decrease in hepatic nuclear factor 6 (Onecut1, 2.23-fold), hepatocyte growth factor (HGF, 1.7-fold), and Stat3 (1.5-fold).

As expected, the adipose tissue of TFA -fed mice contained many more PPAR-related DEGs in response to aspartame and MSG consumption (Figure 1b). The expression of adipose tissue Ppargc1a was reduced by 3.5-fold, together with a 50% reduction in the expression of PPARs α and γ, suggesting significant disruption of mitochondrial biogenesis and lipid metabolism (PPARα and PPARγ, P < 0.05). The transcription of IPA-generated genes involved in the ß-oxidation of fatty acids and glucose were modestly down-regulated, such as rate-limiting enzymes short chain acyl-CoA dehydrogenase (Acads), very long chain acyl-CoA (Acadvl ) and dehydrogenase 3-hydroxyacyl CoA dehydrogenase (Ehhadh) and pyruvate dehydrogenase ß (Pdhb). IPA-generated networks also linked adipogenic gene expression to Ppar transcription factor down-regulation, including increases in Creb1, Crebbp, Notch1 and Lipin1. Additionally, expression of other genes related to lipid mobilization and storage were dysregulated in this model, such as the reverse cholesterol transporter genes Abca1 and Abca3, perilipin5 (Plin5) and apolipoprotein a4 (Apoa4).

Oxidative stress-responsive sirtuins 1 and 2 (Sirt1 and Sirt2) gene were upregulated by 2.4 - 2.6-fold, together with a modest but significant increase in the transcription factor forkhead box 3 (Foxo3) and its target genes: Cdkn1b (p27^Kip1^: 1.41-fold), growth arrest and DNA damage-inducible protein 45 (GADD45b: 1.88-fold), and pro-apoptotic Trp53 (4.4-fold). This was accompanied by a 2.5-fold reduction in p53-binding and regulatory protein Mdm2, and a 1.7-fold reduction in peroxiredoxin 5 (Prdx5), known to protect against oxidative stress. Since PPARs also play an essential role in adipogenesis, we were interested to find changes in gene expression relating to adipocytes growth and differentiation in *Trans* fat-fed mice consuming both food additives. Expression of oncogenic Notch1 was increased by 2.27 fold; which was accompanied by a 1.8-fold increase in nuclear receptor co-repressor 1 (Ncor1) and a 1.6-fold increase in histone deacetylase 1 (Hdac1) expression, suggesting a significant upregulation of this evolutionary conserved intracellular signaling pathway. Furthermore levels of a novel adipocyte differentiating gene mediator (Med23) were increased nearly 3-fold.

The PPAR -downstream adipokine Thrombospondin-1 (Thbs1) was strongly activated in ASP + MSG-fed mice by 4.6-fold, suggesting the possibility of the development of inflammation in these tissues. Serum levels of TNFα were elevated by almost 3-fold in ASP + MSG-fed mice, however we did not detect a significant increase in adipose tissue TNFα gene expression. Further examination of adipose tissue gene expression revealed several important inflammatory genes which were not included in the adipose tissue PPAR networks, including a 3-fold increase in colony stimulating factor Csf1, and pro-inflammatory Interleukin-6 receptor. Expression of 12/15-lipoxygenase (Alox15), key regulator of pro-inflammatory cytokines and chemokines was upregulated by more than 3-fold; accompanied by 3-fold increases in macrophage-marker Emr1. Taken together, the PPAR-related gene networks generated here in response to aspartame and MSG consumption predominantly indicate transcriptional activity related to adipogenesis, ß-oxidation, peroxisomal and mitochondrial function, tissue matrix remodeling, lipid storage and mobilization, and inflammatory mechanisms.

### Coordinated dysregulation of adipose tissue genes involved in glucose and lipid catabolism and ß-oxidation in ASP + MSG-treated mice

The highest levels of adipose tissue, glucose and plasma free fatty acids were apparent in *Trans* fat-fed mice consuming both ASP and MSG; and since analysis of PPAR-related gene networks revealed diet-induced changes in the expression of some genes involved in fatty acid oxidation, we next focused more specifically on differentially expressed genes associated with lipolysis, glycolysis, the citric acid cycle and mitochondrial oxidative phosphorylation (Ox-Phos), together with other genes with mitochondrial and peroxisomal biogenesis functions. Table 3 shows fold-changes in the expression of 60 genes with these classifications, which were dysregulated by the combination of ASP + MSG by ≥1.40-fold, and indicates that 50% of these were also dysregulated by ASP alone. For example in adipose tissue but not liver, we found significantly attenuated expression of both short-chain and very long-chain Acyl-coA dehydrogenase, which catalyze the first steps in the oxidation of free fatty acids. Expression of the trifunctional enzyme subunit Hydroxyacyl-CoA dehydrogenase/3-ketoacyl-CoA thiolase/enoyl-CoA hydratases α and ß (Hadha and Hadhb), which catalyzes the last three steps of mitochondrial ß-oxidation of long chain fatty acids was also significantly attenuated in *Trans* fat-fed mice consuming a combination of ASP and MSG. In addition to the 50% reduction in expression of adipose tissue PPARα, ASP + MSG-fed mice exhibited a significant reduction in Enoyl-CoA, hydratase/3-hydroxyacyl CoA dehydrogenase (Ehhadh), the second enzyme in the inducible peroxisomal ß-oxidation pathway. Glycolysis activator genes 6-phosphofructo-2-kinase/fructose-2,6-biphosphatase 2 (Pfkfb2 and Pfkfb4) were upregulated just over 2-fold.

**Table 3:** Diet-specific coordinated dysregulation of adipose tissue genes involved in glucose and lipid catabolism expressed as significant fold changes (P < 0.05, stringency ≥ ± 1.4-fold change in expression)

	**Symbol**	**Induced by MSG**	**Induced by ASP**	**Induced by ASP + MSG**	**Sig.**
**Mitochondrial ß-oxidation genes**					
Peroxisome proliferator-activated recepto rγ, coactivator 1α	Ppargc1a	−1.94	−4.51	−3.49	0.048
Peroxisome proliferator-activated receptor α	Ppara	−1.01	−1.99	−2.03	0.052
Carnitine palmitoyltransferase 1B	Cpt1b	−1.99	−1.46	−1.63	0.028
Acyl-CoA dehydrogenase, short-chain	Acads	−1.36	−1.32	−1.57	0.024
Acyl-CoA dehydrogenase, very long chain	Acadvl	−1.32	−1.11	−1.67	0.036
Hydroxyacyl-CoA dehydrogenaseα	Hadha	−1.14	−1.31	−1.50	0.036
Hydroxyacyl-CoA dehydrogenase ß	Hadhb	−1.06	−1.43	−1.73	0.051
**Citric acid cycle (TCA) genes**					
Aconitase 1, soluble	Aco1	−1.79	−1.69	−1.67	0.003
Isocitrate dehydrogenase 2 (NADP+), mitochondrial	Idh2	−1.19	−1.36	−1.44	0.022
Pyruvate dehydrogenase (lipoamide) α2	Pdha2	−1.43	−1.82	−2.36	0.004
Pyruvate dehydrogenase (lipoamide) ß	Pdhb	−1.30	−1.36	−1.62	0.043
Succinate dehydrogenase complex, subunit B	Sdhb	−1.32	−1.30	−1.44	0.001
**Mitochondrial oxidative phosphorylation genes**					
NADH dehydrogenase (ubiquinone) 1 α subcomplex 5	Ndufa5	−1.14	−1.19	−1.47	0.007
NADH dehydrogenase (ubiquinone) 1 α subcomplex 12	Ndufa12	−1.21	−1.24	−1.46	0.004
NADH dehydrogenase (ubiquinone) 1ß subcomplex 2	Ndufb2	−1.12	−1.34	−1.55	0.016
NADH dehydrogenase (ubiquinone) 1ß subcomplex 6	Ndufb6	−1.11	−1.31	−1.46	0.050
NADH dehydrogenase (ubiquinone) 1ß subcomplex10	Ndufb10	1.01	−1.20	−1.76	0.010
NADH dehydrogenase (ubiquinone) Fe-S protein 1	Ndufs1	−1.62	−1.46	−2.16	0.006
NADH dehydrogenase (ubiquinone) Fe-S protein 6	Ndufs6	−1.29	−1.38	−1.53	0.037
NADH dehydrogenase (ubiquinone) flavoprotein 1	Ndufv1	−1.16	−1.27	−1.60	0.005
Succinate dehydrogenase complex, subunit B	Sdhb	−1.32	−1.30	−1.44	0.001
Ubiquinol-cytochrome c reductase, complex 3 subunit 6	Uqcr11	−1.44	−1.44	−1.58	0.006
COX assembly mitochondrial protein homolog	Cmc1	−1.17	−2.33	−2.53	0.040
Cytochrome c oxidase subunit 7a polypeptide 1	Cox7a1	−1.34	−1.25	−1.66	0.005
Cytochrome c oxidase, subunit 7b	Cox8b	−1.32	−1.13	−1.58	0.047
Cytochrome c oxidase assembly homolog	Cox11	−1.04	−1.32	−1.46	0.024
COX assembly mitochondrial protein homolog	Cmc1	−1.17	−2.33	−2.53	0.040
Uncoupling protein 2 (mitochondrial, proton carrier)	Ucp2	1.32	1.43	1.59	0.001
**Glycolysis**					
6-phosphofructo-2-kinase/fructose-2,6-biphosphatase 2	Pfkfb2	1.37	1.99	2.47	0.050
6-phosphofructo-2-kinase/fructose-2,6-biphosphatase 4	Pfkfb4	1.53	2.48	2.85	0.001
**Mitochondrial biogenesis & assembly**					
Polymerase gamma (nuclear encoded)	Polg	1.42	1.72	1.94	0.038
Transcription factor A, mitochondrial	Tfam	−1.38	−1.32	−2.07	0.008
Mitochondrial fission process 1	Mtfp1	−1.37	−1.30	−1.64	0.004
Tu translation elongation factor, mitochondrial	Tufm	−1.07	2.14	1.68	0.011
G elongation factor, mitochondrial 2	Gfm2	−1.22	−2.30	−2.46	0.034
Mitochondrial ribosomal protein L3	Mrpl3	−1.06	2.03	1.79	0.054
Mitochondrial ribosomal protein L20	Mrpl20	−1.21	−1.41	−1.62	0.024
Mitochondrial ribosomal protein S24	Mrps24	1.53	−2.99	−3.31	0.037
Mitochondrial ribosomal protein S7	Mrps7	−1.12	−1.39	−1.63	0.032
Mitochondrial ribosomal protein S12	Mrps12	−1.36	−1.40	−1.73	0.011
Mitochondrial ribosomal protein S27	Mrps27	−1.16	−1.28	−1.55	0.050
Surfeit 1	Surf1	−1.22	−1.46	−1.57	0.026
Mitochondrial inner membrane organizing system 1	Minos1	−1.02	−1.44	−1.50	0.001
Inner membrane protein, mitochondrial	Immt	−1.13	−1.33	−1.66	0.009
Peroxiredoxin 5	Prdx5	−1.15	−1.51	−1.70	0.029
DnaJ (Hsp40) homolog, subfamily C, member 27	Dnajc27	−1.48	−1.80	−1.51	0.047
Diablo, IAP-binding mitochondrial protein	Diablo	1.14	1.51	1.63	0.007
**Mitochondria-dependent apoptosis**					
BCL2-associated X protein	Bax	1.23	1.38	1.66	0.032
Tumor protein p53	Trp53	2.13	3.57	4.39	0.004
Caspase 9	Casp9	1.44	1.86	1.70	0.034
E2F transcription factor 1	E2f1	1.52	1.73	2.60	0.025
**Peroxisomal genes**					
Peroxisomal biogenesis factor 1	Pex1	−1.14	−2.02	−2.05	0.054
Peroxisomal biogenesis factor 5	Pex5	−1.29	−1.55	−1.67	0.039
Peroxisomal biogenesis factor 7	Pex7	−1.10	−1.74	−1.46	0.026
Peroxisomal biogenesis factor 11 α	Pex11a	−1.11	−1.40	−1.48	0.012
Peroxisomal biogenesis factor 13	Pex13	−1.17	−1.60	−1.43	0.055
Enoyl-CoA, hydratase/3-hydroxyacyl CoA dehydrogenase	Ehhadh	−1.40	−1.06	−1.55	0.029
Acetyl-CoA acyltransferase 1	Acaa1a	−1.32	−1.35	−1.59	0.056
ATP-binding cassette, sub-family D (ALD), member 1	Abcd1	1.29	1.69	2.09	0.051
Epoxide hydrolase 2	Ephx2	−1.17	−1.62	−1.66	0.010
**Calcium homeostasis genes**					
Calcium homeostasis endoplasmic reticulum protein	Cherp	1.55	2.21	2.37	0.002
Calcium homeostasis modulator 2	Calhm2	1.19	1.61	2.09	0.001
Calcium activated nucleotidase 1	Cant1	1.16	1.45	1.86	0.002
Mitochondrial calcium uniporter	Ccdc109a	1.11	1.44	1.70	0.047
Calcium channel, voltage-dependent, P/Q type, alpha 1A subunit	Cacna1a	1.37	1.65	1.62	0.016
S100 calcium binding protein A8	S100a8	−1.15	−1.60	−1.57	0.033
Mitochondrial calcium uptake 1	Cbara1	−1.08	−1.80	−1.71	0.038
Calcium channel, voltage-dependent, gamma subunit 4	Cacng4	−1.26	−1.63	−1.85	0.000
Calcium binding protein 2	Cabp2	−1.26	−1.71	−1.98	0.015
S100 calcium binding protein A13	S100a13	−1.19	−2.09	−1.99	0.039
Solute carrier family 8 (sodium/calcium exchanger), member 1	Slc8a1	−1.40	−2.28	−2.01	0.042
Calcium channel, voltage-dependent, gamma subunit 8	Cacng8	−1.21	−1.87	−2.13	0.026
Calcium channel, voltage-dependent, T type, alpha 1I subunit	Cacna1i	−1.21	−1.38	−2.61	0.045
Calcium channel, voltage-dependent, beta 2 subunit	Cacnb2	−1.56	−3.66	−2.76	0.037
Calcium channel, voltage-dependent, alpha 2/delta subunit 4	Cacna2d4	−1.77	−3.32	−3.35	0.019
Calreticulin 3	Calr3	−1.19	−1.83	−1.71	0.035

Lastly, the expression of genes involved in mitochondrial and peroxisomal biogenesis and apoptosis were affected by food additive consumption, including a 50% reduction in expression of mitochondrial transcription factor A (Tfam), a key activator of mitochondrial transcription as well as a participant in mitochondrial genome replication. Conversely, an increase in mitochondrial apoptosis was suggested by the upregulation of p53, E2F transcription factor 1, caspase 9 and Bax. In the liver, relatively few lipid catabolic genes were significantly down-regulated compared to the adipose tissue; conversely some important ß-oxidative genes such as Cpt1c were modestly upregulated.

### Expression of food additive-induced DEGs common to both liver and adipose tissue

**Figure 2:**
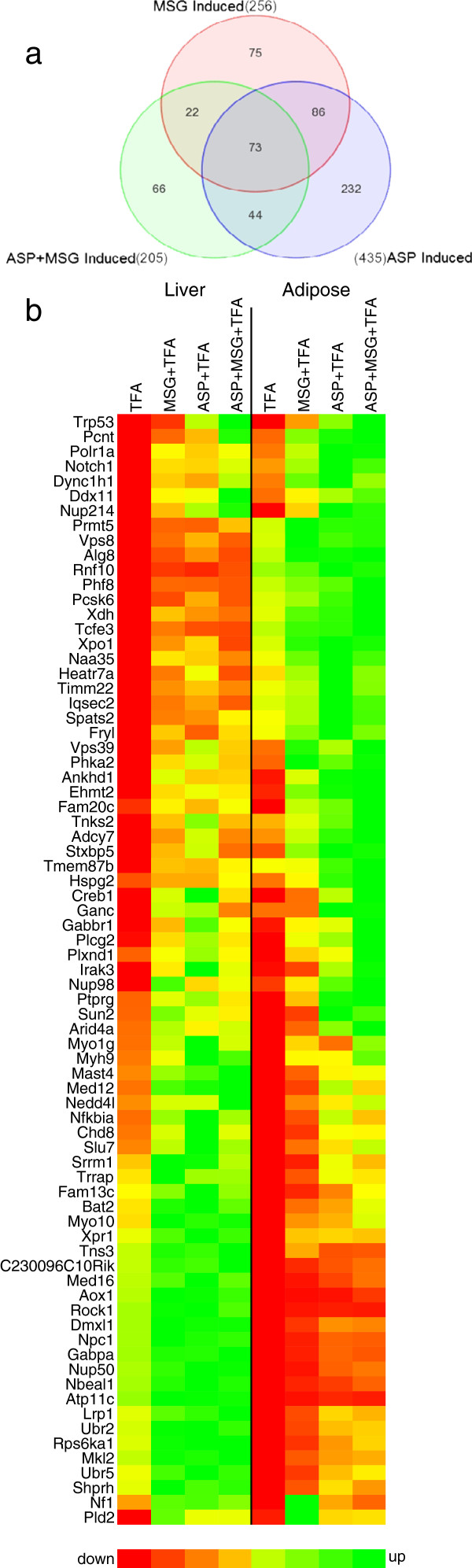
**Upregulated gene expression shared by liver and adipose tissue. (a)** Venn diagram displaying numbers of upregulated DEGs common to both liver and adipose tissue induced by either ASP, MSG or the combination of both additives. **(b)** 2-dimentional heat map of 73 upregulated DEGs common to both hepatic and adipose tissue (P < 0.05, stringency ≥ ± 1.4-fold change in expression).

**Figure 3:**
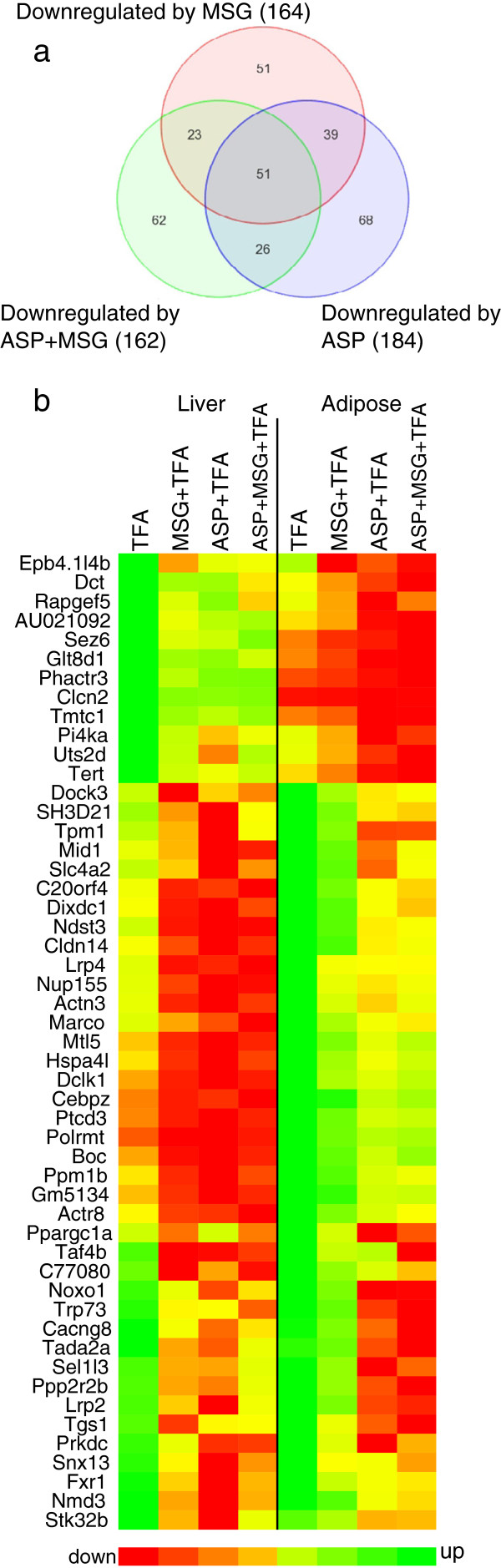
**Downregulated gene expression shared by liver and adipose tissue. (a)** Venn diagram displaying numbers of downregulated DEGs common to both liver and adipose tissue induced by either ASP, MSG or the combination of both additives. **(b)** 2-dimentional heat map of 51 downregulated DEGs common to both hepatic and adipose tissue (P < 0.05, stringency ≥ ± 1.4-fold change in expression).

Our data show that the expression of many genes related to fatty acid catabolism where significantly down-regulated in the adipose tissue, but to a far lesser extent in the liver. The final part of our analysis aimed to shed more light on the nature of genes which were differentially regulated by the additives aspartame and MSG in both the liver and the adipose tissue of *Trans* fat-fed mice, in order to further our understanding of the mechanism(s) involved in the dyslipidemia and other metabolic disturbances. Figures 2 and 3 show Venn diagrams (A) and 2-D heat-maps (B) of sub-lists of DEGs considered significant only if they were up-regulated by ≥1.4-fold in response to ASP, MSG or a combination of the two additives, in both the liver and adipose tissue (Figure 2, P < 0.05); or down-regulated by the same criteria (Figure 3, P < 0.05). Almost twice as many genes were up-regulated than down-regulated in both tissues by the food additives, either alone (i.e. comparison ASP + TFA versus TFA, and comparison MSG + TFA versus TFA) or in combination (comparison ASP + MSG + TFA versus TFA). A total of 598 DEGs were up-regulated in all 3 comparisons, compared to only 320 down-regulated DEGs. Interestingly, aspartame alone induced the highest number of upregulated DEGs common to both tissues; followed by MSG; and the lowest number of upregulated DEGs was induced by the combination of both additives. A total of 73 DEGs common to both hepatic and adipose tissue were upregulated in response to a combination of ASP + MSG, and only 51 commonly expressed hepatic and adipose tissue DEGs were downregulated. Most notably up-regulated were a number of stress-responsive DEGs including Trp53 (p53), Notch1, phospholipase D2 (Pld2) and xanthine dehydrogenase (Xdh). Stress / remodeling proteoglycan Perlecan (Hspg2) was robustly up-regulated together with LDL-receptor related protein 1 (Lrp1), Plexin D1 (Plxnd1), Rock1, and Neurofibromatosis 1 (Nf1); all suggesting that food additive consumption may have led to an upregulation of genes involved in stress, proliferation, vasculogenesis and angiogenesis in both tissues (Figure 2b, P < 0.05).

**Figure 4:**
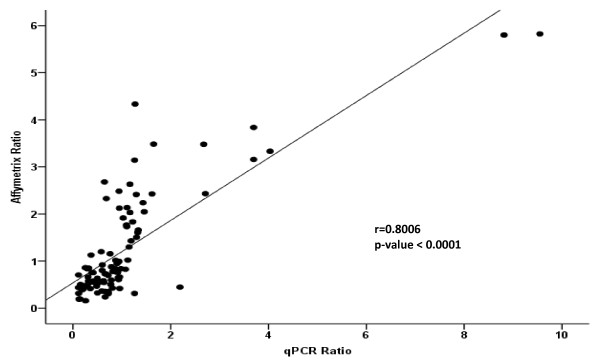
**Correlation of qPCR and microarray data.** Scatter plot shows Correlation of the ratios from the microarray and real-time PCR data set. Genes that differed significantly (P < 0.01) in their regulation between the diet groups’ microarray analysis were selected and validated with the same samples by qPCR analysis. Ratios of expressions between the diet comparisons calculated from the microarray data set correlated well with the ratio calculated from the qPCR data (r = 0.641, P < 0.0001). A complete list of these genes is shown in Additional file 2.

Key transcription factor Ppargc1a was significantly down-regulated in both tissues but particularly in adipose (Figure 3b); which was accompanied by a 2-fold reduction in hepatic expression of the Cl(−)/HCO [3] (−) anion exchanger 2 (Slc4a2), which is critically involved in the development of biliary cirrhosis. Two members of endocytic low density lipoprotein-related protein family (Lrp) were also down-regulated in both adipose and hepatic tissues. Expression of Lrp2, otherwise known as Megalin, was decreased 3-fold in adipose and 1.7-fold in liver tissue; whereas functionally-related Lrp4 expression was halved in both tissues. We also found a significant reduction in the expression of NADPH oxidase 1 involved in the regulation of superoxide generation, particularly in ASP + TFA diet comparisons. Fourteen genes were randomly chosen for further analyzed by qPCR based on biological relevance. Pearson correlation coefficients between the microarray analysis and qPCR were calculated. Ratios of expressions between the diet comparisons calculated from the microarray data set correlated well with the ratio calculated from the real-time PCR data (Figure 4, r = 0.641, P < 0.0001). A complete list of these genes and PCR primers is given in Additional file 2.

Taken together our final analysis suggests that the transcription of a number of functionally related hepatic and adipose tissue genes and transcription factors can be modulated in a similar manner by food additive consumption. These results may allow for informed speculation about the mechanisms behind the additive-induced metabolic dysregulation seen in this nutrigenomics model.

## Discussion

Our present data suggests that exposure to the food additives aspartame (ASP) and monosodium glutamate (MSG) as part of a *Trans* fat-enriched diet may cause metabolic dysregulation and alterations in hepatic and adipose tissue gene expression. Exposure to these additives offered continuously in the drinking water either alone or in combination, commenced *in utero* via the mother’s diet and continued throughout the first five months of life. These metabolic changes were not incurred by hyperphagia since all four diet groups ingested equal amounts of food. We have previously shown that ASP exposure in C57Bl/6 J mice may promote weight gain and dysregulation of glucose homeostasis when consumed together with a standard chow diet (typically 5% dietary lipids) [38]. We have also demonstrated that ASP may interact with a second widely consumed food additive MSG to further imbalance glucose homeostasis in standard chow-fed animals [25]. The level of MSG-exposure in our model (~130 mg/Kg bw) is considerably lower than the dosage used in the classic non-genetic MSG-obese model, in which animals are typically injected with 4 g/Kg bw MSG for a period of several days shortly after birth when the blood–brain-barrier (BBB) is still immature and vulnerable to excitotoxic damage [14-16,21]. Nevertheless, exposure to MSG and ASP metabolites in our model began during conception and continued *in utero* and throughout life; and we hypothesize that it is this continuous exposure which resulted in the metabolic imbalances described herein.

Our early nutrigenomics studies additionally focused upon lipogenic changes in hepatic and adipose tissue gene expression incurred as a result of consumption of *Trans* Fatty Acid (TFA), either alone or in conjunction with MSG [9]. *Trans* fats are unsaturated fatty acids with *trans* double bonds introduced during the catalytic partial hydrogenation process in order to stabilize dietary fats and prolong their shelf-life. However, an insightful study by Yu *et al.*[39] showed that some TFAs such as elaidic acid are incompletely oxidized in mitochondria due to the fact that one of the metabolites (5-*trans*-tetradecenoyl-CoA) is a poorer substrate for the mitochondrial enzyme long chain acyl-CoA dehydrogenase (Acadl) than its *cis* isomer 5-*cis*-tetradecenoyl-CoA. Elaidic acid is the major fat found in hydrogenated vegetable oils, and a reduction in the oxidative capacity of mitochondria for the *trans* isomer of the elaidic acid intermediate may result in an accumulation of 5-*trans*-tetradecenoyl-CoA in the mitochondrial matrix, which could potentially increase oxidative stress. In our previous study [9], TFA-feeding robustly upregulated lipid biosynthetic enzymes in the adipose tissue and liver whilst also promoting increases in the transcription of mitochondrial uncoupling protein 3 (Ucp3), several mitochondrial ß-oxidation genes encoding short-, medium-, long- and very long-chain acyl co-A dehydrogenases, together with the citric acid cycle genes isocitrate dehydrogenase 3 (Idh3) and pyruvate dehydrogenase complex (Pdhx). The mechanism for this appeared to involve a 50% reduction in the expression of the transcription factor Ppargc1a, the master homeostatic transcriptional controller of mitochondrial biogenesis, oxidative phosphorylation and energy metabolism [40]. Interestingly, the addition of MSG further reduced the expression of adipose tissue Ppargc1a to 25% of control levels, together with attenuated expression of Idh3, Pdhx, Ucp3, and several ß-oxidation genes [9]. In the present study, we sought to extend our investigation of hepatic and adipose tissue gene expression to include the commonly-consumed food additive ASP, since we have previously shown that ASP may interact with MSG *in vivo* to alter glucose homeostasis, and because others have shown that ASP may disrupt hepatic antioxidant defenses [24].

In TFA-fed mice, the addition of either ASP or MSG alone did not increase adiposity, fasting serum glucose, cholesterol or FFA levels. However in combination, ASP + MSG significantly elevated all these parameters, and doubled fasting serum leptin and TNFα, whilst promoting insulin resistance and hepatic steatosis. These obesogenic changes were accompanied by striking alterations in gene transcription, most notably in the adipose tissue deposits which were significantly larger than those in the additive-free TFA diet group. This is of interest since white adipose tissue (WAT) was originally considered to be a passive depot of triglycerides, transcriptionally inert and sequestering or releasing fatty acids under the influence of various hormones [41]. However the past 15 years of research have shown that WAT is a dynamic pleiotropic endocrine organ responsive to a wide array of chemical, hormonal, neuronal and environmental signals. In TFA-fed mice, we identified over 11,000 differentially expressed adipose transcripts affected by exposure to ASP and MSG commencing *in utero,* and roughly 8,000 in liver. Others have also shown higher numbers of adipose tissue differentially expressed genes (DEGs) compared to liver, both in response to fasting [42], and during LPS-induced inflammation [43]. Most recently, high-fat feeding has been shown to induce obesogenic gene expression changes in 4 murine tissues, with adipose tissue producing the second highest number of DEGs in the following order: skeletal muscle > adipose > liver > heart [44].

The increased hepatic triglyceride content seen in TFA-fed mice exposed to both MSG-containing diets was accompanied by an increase in the expression of acetyl-CoA carboxylase ß (Acc2) and carnitine palmitoyltransferase 1 (Cpt1). Previous studies have shown that transcription of hepatic Acc2 is markedly upregulated by increased food intake [45], insulin, and dexamethasone [46]. Unlike Acc1 which stimulates lipogenesis, Acc2 is known to inhibit lipolysis; and its structure has an N-terminal extension containing a mitochondrial-targeting motif which allows Acc2 to associate with Cpt1 on the outer mitochondrial membrane, thus regulating ß-oxidation at an early stage in the process [44]. High-fat diets and obesity have previously been associated with decreased expression of Ppargc1a, the dominant regulator of oxidative metabolism [41]; and whereas we have previously shown that Ppargc1a expression is reduced by intake of TFA compared to low-fat controls [9], we now demonstrate that levels of this transcription factor can be further attenuated by ASP + MSG exposure, both in the liver and particularly in the visceral adipose tissue. Expression of Ppargc1a is reduced in insulin resistant tissues [40], during obesity [47] and in type 2 diabetes [48]; and Ppargc1a knock-out mice exhibit hepatic steatosis due to a reduction in mitochondrial oxidative capacity and mtDNA content, together with an upregulation of lipogenic gene expression [49]. A large number of signaling pathways have been proposed to regulate Ppargc1a, including thyroid hormone, nitric oxide synthase, p38 mitogen activated protein kinase (p38MAPK), sirtulin and β-adrenergic stimulation, to name but a few [49]. Interestingly, in addition to the reduced expression of Ppargc1a in obese subjects [47], sustained exposure to saturated fatty acids has been shown to attenuate the expression of Ppargc1a *in vitro*[50]; and epigenetic studies have shown a negative correlation between Ppargc1a mRNA content and an increase in the methylation of the promoter region of Ppargc1a in the skeletal muscle of patients with type 2 diabetes [51].

In broad terms, diet-induced changes in hepatic gene expression tended to be less extensive, and did not mirror the coordinated reduction in adipose tissue ß-oxidative gene expression. Reduced Ppargc1a expression was accompanied by an upregulation of genes involved in lipid and cholesterol metabolism, cell stress adaptation and hepatoprotection. Additionally, levels of carnitine palmitoyltransferase (Cpt1c), an essential enzyme in the ß-oxidation of long-chain fatty acids was modestly elevated in the livers of MSG and ASP-treated mice, together with genes involved in lipid and cholesterol homeostasis. Markers of significant hepatic microsteatosis and oxidative damage have previously been noted in the livers of MSG-treated rodents [21,52,53]; some of which could be partially attenuated with antioxidant vitamins C [54], E [55] or both [56]. Long-term ASP exposure has also been associated with diminished liver function and impaired antioxidant defenses [24]. The authors of that study speculated that the hepatotoxicity could be attributed to the methanol component of metabolized ASP, since methanol is a well characterized hepatotoxin [57]. However in our model the relatively small amount of ASP offered in the diet would have been insufficient to raise blood methanol levels to that required to promote hepatotoxicity; rather, the increased expression of genes with hepatoprotective ontology may have been deregulated due to the increased hepatic triglyceride and serum FFA incurred by neonatal exposure to the combination of ASP and MSG.

Although our nutrigenomic evidence points to significant differences in the ontology of gene expression between the adipose tissue and the liver, for example in the differential expression of ß-oxidative genes; there were a number of common DEGs which were up- or down-regulated similarly in both tissues, and may shed more light on the mechanism for the diet-induced metabolic dysregulation. Some of the most interesting were adaptogenic, stress-responsive and oxidative DEGs such as p53, Notch1, Ppargc1a, NADPH oxidase organizer 1(Noxo1) and xanthine dehydrogenase. The transcriptional activation of p53 facilitates a diverse range of responses to metabolic challenges including the regulation of glucose homeostasis, substrate oxidation and OX-PHOS, mitochondrial integrity, autophagy and apoptosis. This assists in balancing proliferation and growth with nutrient availability, whilst attenuating metabolic stress-induced damage. In the liver, activation of p53 enhances apoptosis and promotes NAFLD-associated insulin resistance [58]; whilst in the adipose tissue, p53 has been termed the “guardian of corpulence” for its adaptogenic role in lipogenesis and in protecting against FFA-induced ROM and cellular stress [59]. Concomitant with the ASP + MSG-induced increase in p53 expression, we also noted a decrease in one of the major p53 binding and regulating proteins, Mdm2. This protein attaches to p53, promoting destabilization and removal from the nucleus, inhibiting its ability to activate transcription [60]. Thus our data supports the notion that there is generally an inverse relationship between Mdm2 and p53 expression via a negative feedback loop [61], suggesting that the diet-induced reduction in Mdm2 assists in enhancing the activation of p53. In some tissues p53 has been shown to interact with Notch1, a trans-membrane receptor molecule participating in cell to cell signaling and which has previously been implicated in the control of adipogenesis [62]. We observed a diet-induced increase in the expression of Notch1 in both the adipose tissue and the liver, and whereas the role of notch1 in tumorigenesis has been well documented [63], recent evidence also suggests that notch1 may play a role in the development of insulin resistance [64] and may also have several other pleiotropic functions in the liver [65].

## Conclusion

The extent of visceral and hepatic fat accumulation and gene expression can be significantly modified by exposure to common food additives consumed as part of a *Trans* fat diet. The combination of aspartame and MSG promoted the highest level of visceral fat deposition and serum FFA, leptin and TNFá compared to control or either additive alone. Further studies are warranted to examine the effect of food additives, and combinations of additives on obesogenic gene expression.

## Competing interests

All authors declare that they have no conflict of interest and have not received funding from Industry sources.

## Authors’ contributions

KSC conceived the study, designed the experiments and drafted the manuscript. NJM, AI, BLA, SS and RU performed the experiments. MZZ, KSC and NJM analyzed the data. FAA & MZ helped draft the manuscript. We are grateful for continuous discussions and support from FAA. All Authors read and approved the final manuscript.

## Supplementary Material

Additional file 1 Diet composition. Click here for file

Additional file 2 qPCR Primer sequences used in the confirmatory analysis. Click here for file

Additional file 3 **A: Diet groups and experimental design.** B and C: 3-D heat maps of standardized expression values of 10,117 DEGs in liver (**B**) and 28,101 DEGs in adipose tissue (**C**), with ANOVA p-value of <0.05 for diet. Click here for file

Additional file 4 **Histological examination of hepatic tissue stained with H&E (A: x40 magnification); and adipose tissue stained with trichrome (B: x20 magnification).** Click here for file

Additional file 5 Diet-specific differences in the number of significant DEGs dysregulated by MSG, ASP, or the combination of ASP + MSG in liver and adipose tissue. Click here for file

Additional file 6 **Diet-specific differences in the functional ontology of genes dysregulated by dietary MSG (A, B); ASP (C, D) and the combination ASP + MSG (E, F).** Click here for file
