# Supplementary material for: Prediabetic changes in gene expression induced by aspartame and monosodium glutamate in Trans fat-fed C57Bl/6 J mice
Source: Nutr Metab (Lond). 2013 Jun 19;10:44. doi: 10.1186/1743-7075-10-44 (PMC3727955; doi:10.1186/1743-7075-10-44)
Supplement: Additional file 1 — Diet composition. [file 1743-7075-10-44-S1.pdf]

Diet composition

**Ingredients (g/100g dietary weight)**	**TFA**	**ASP+TFA**	**MSG+TFA**	**ASP+MSG+TFA**
Protein (%)	19.1	19.1	19.1	19.1
Carbohydrate (%)	37.7	37.7	37.7	37.7
Fat (%) (Ether extract)	28	28	28	28
Fiber	4.3	4.3	4.3	4.3
Vitamins, Minerals & Ash	10.9	10.9	10.9	10.9
Energy (kcal/g)	4.39	4.39	4.39	4.39
ASP (mg/kg BW)	0	43.54	0	43.54
MSG (mg/kg BW)	0	0	130.03	130.03
