# Supplementary material for: Prediabetic changes in gene expression induced by aspartame and monosodium glutamate in Trans fat-fed C57Bl/6 J mice
Source: Nutr Metab (Lond). 2013 Jun 19;10:44. doi: 10.1186/1743-7075-10-44 (PMC3727955; doi:10.1186/1743-7075-10-44)
Supplement: Additional file 2 — qPCR Primer sequences used in the confirmatory analysis. [file 1743-7075-10-44-S2.pdf]

Table: PCR primers for qRT-PCR

Gene Name	Gene Symbol	Accession number	Forward primer	Reverse primer
Tumor protein p53	Trp53	NM_011640	5' CCG CCG TAC AGA AGA AGA AA 3'	5' GCG GAT CTT GAG GGT GAA ATA 3'
Peroxisome proliferator-activated receptor gamma, CoAα	Ppargc1a	NM_027710	5' AGC TCC AAG ACC AGG AAA TC 3'	5' GAA GTC GCC ATC CCT TAG TT 3'
Thrombospondin 1	Thbs1	NM_011580	5' CAA TTT TCA GGG GGT GCT GC 3'	5' CCG TTC ACC ACG TTG TTG TC 3'
Transcription factor A, mitochondrial	Tfam	NM_009360	5' CCC CTC GTC TAT CAG TCT TGT C 3'	5' TTC TGG TAG CTC CCT CCA CA 3'
Collagen, type I, alpha 2	Col1a2	NM_007743	5' AGT CGA TGG CTG CTC CAA AA 3'	5' AGC ACC ACC AAT GTC CAG AG 3'
Enoyl-CoA, hydratase/3-hydroxyacyl CoA dehydrogenase	Ehhadh	NM_023737	5' CTG GCT ATG ATC CGC CTC TG 3'	5' TCA GCA CCT GCA CAG AAG TT 3'
Arachidonate 15-lipoxygenase	Alox15	NM_009660	5' CTG GAT GAG GAG CTC AAG AAA G 3'	5' CCA GGT ACT GCT GAC TAC AAA G 3'
Fibronectin 1	Fn1	NM_010233	5' TCC TGT CTA CCT CAC AGA CTA C 3'	5' GTC TAC TCC ACC GAA CAA CAA 3'
Glutamate receptor, ionotropic, N-methyl D-aspartate 1	Grin1	NM_008169	5' ACT CCC AAC GAC CAC TTC AC 3'	5' GTA GAC GCG CAT CAT CTC AA 3'
Glutamate receptor, ionotropic, N-methyl D-aspartate 2c	Grin2c	NM_010350	5' GCA GAA CTT CCT GGA CTT GC 3'	5' CAC AGC AGA ACC TCC ACT GA 3'
Glutamate receptor, ionotropic, N-methyl D-aspartate 2a	Grin2a	NM_008170	5' AGA CCT TAG CAG GCC CTC TC 3'	5' CTC TTG CTG TCC TCC AGA CC 3'
Glutamate receptor, ionotropic, kainate 3	Grik3	NM_001081097	5' GGT AGG CCA GCA GAA GAA ATA 3'	5' CGC CAA TGA GTA GAT GGT AGA G 3'
Glutamate receptor, ionotropic, kainate 4	Grik4	NM_175481	5' TAT GTC ATG CCC AGA CCA GC 3'	5' GAT AGC AGC AAT CCT CAG GGA 3'
