# Supplementary figures and images for: Prediabetic changes in gene expression induced by aspartame and monosodium glutamate in Trans fat-fed C57Bl/6 J mice
Source: Nutr Metab (Lond). 2013 Jun 19;10:44. doi: 10.1186/1743-7075-10-44 (PMC3727955; doi:10.1186/1743-7075-10-44)

**Control**

**MSG**

**ASP**

**ASP+ MSG**

**A**

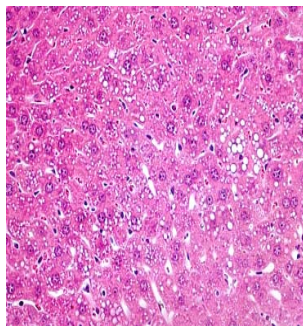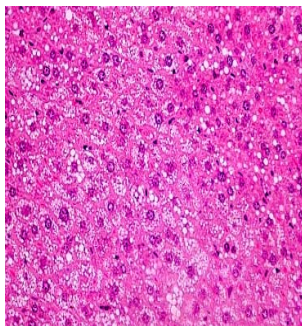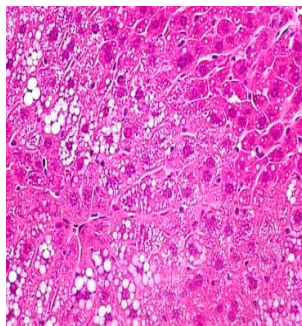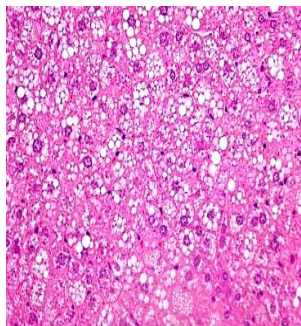

**B**

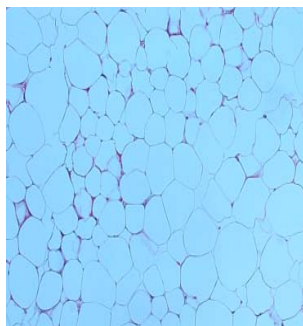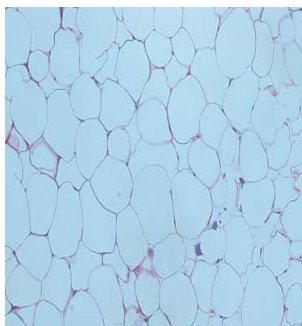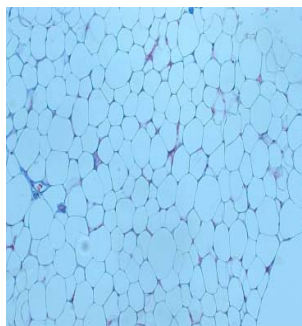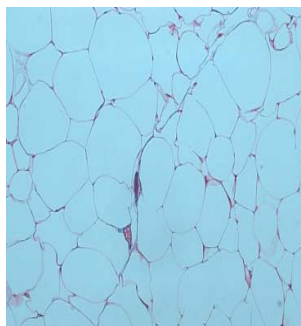

Supplement: Additional file 3 — A: Diet groups and experimental design. B and C: 3-D heat maps of standardized expression values of 10,117 DEGs in liver (B) and 28,101 DEGs in adipose tissue (C), with ANOVA p-value of <0.05 for diet. [file 1743-7075-10-44-S3.pdf]

## LIVER

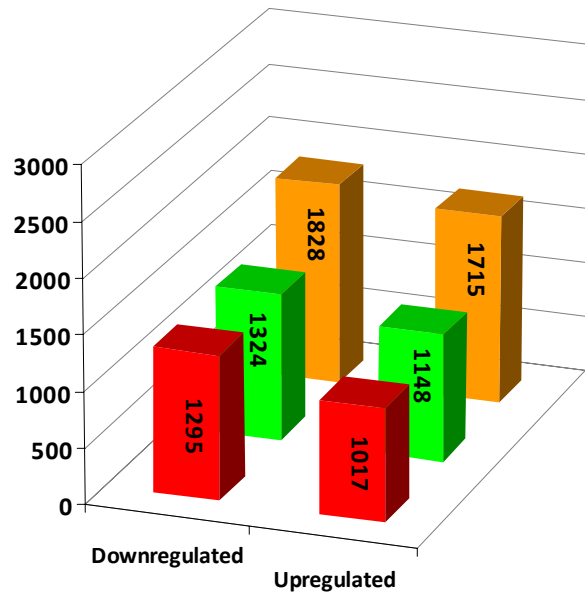

■ MSG INDUCED ■ ASP+MSG INDUCED ■ ASP INDUCED

## ADIPOSE

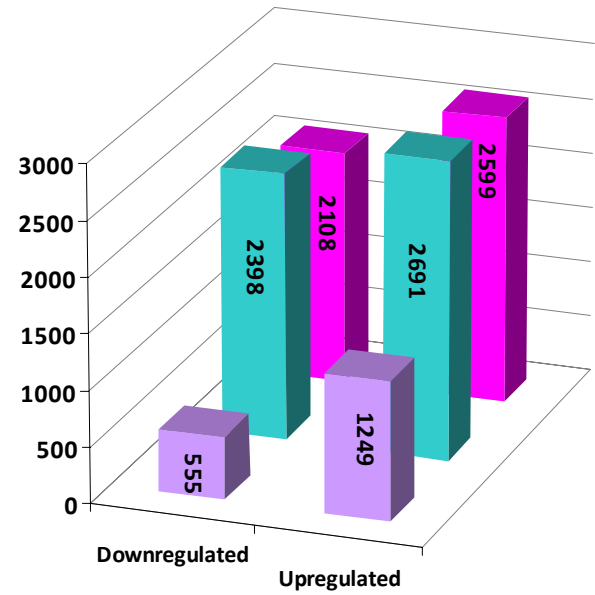

■ MSG INDUCED ■ ASP + MSG INDUCED ■ ASP INDUCED

Supplement: Additional file 4 — Histological examination of hepatic tissue stained with H&E (A: x40 magnification); and adipose tissue stained with trichrome (B: x20 magnification). [file 1743-7075-10-44-S4.pdf]

**A**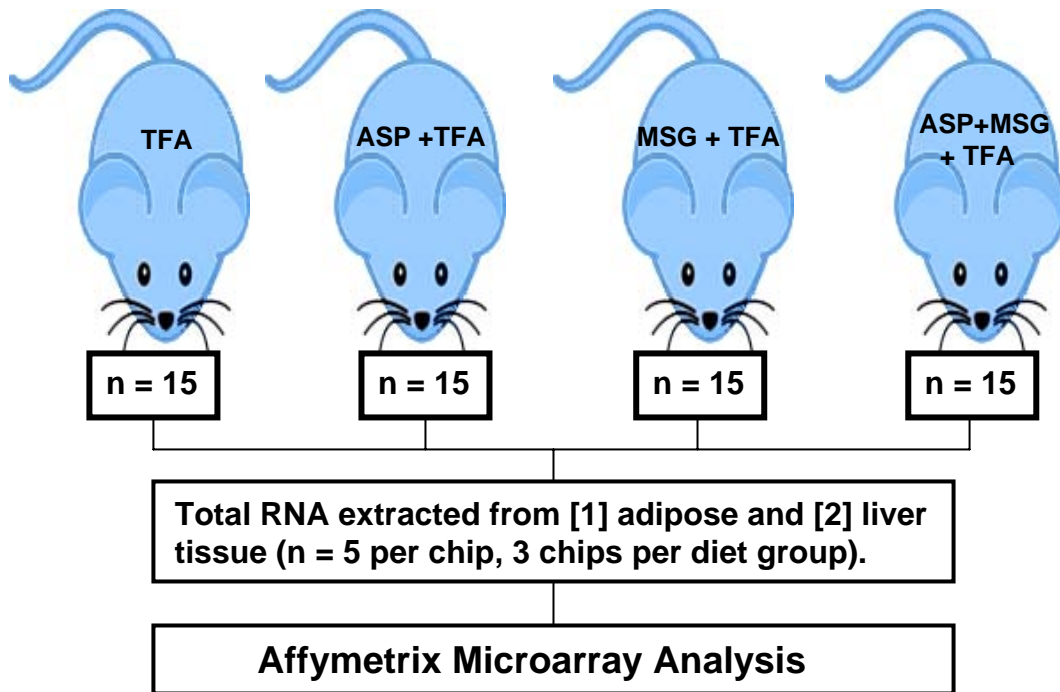**B**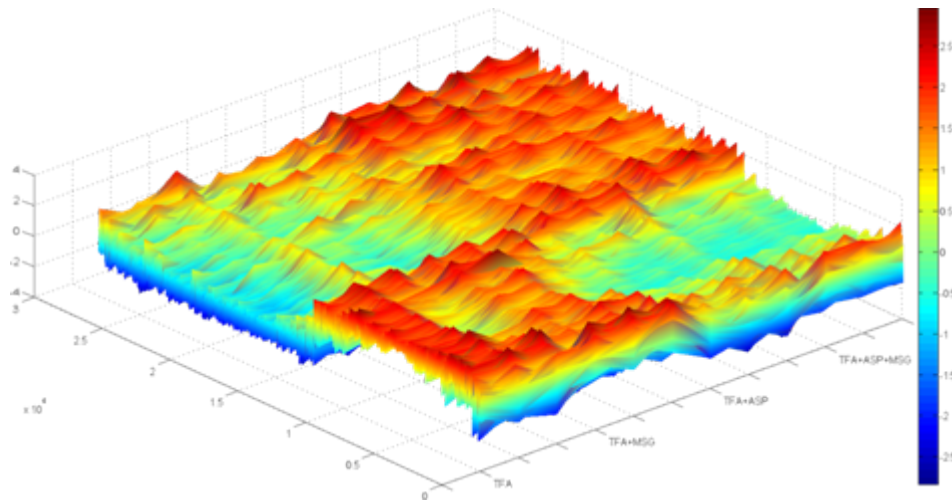**C**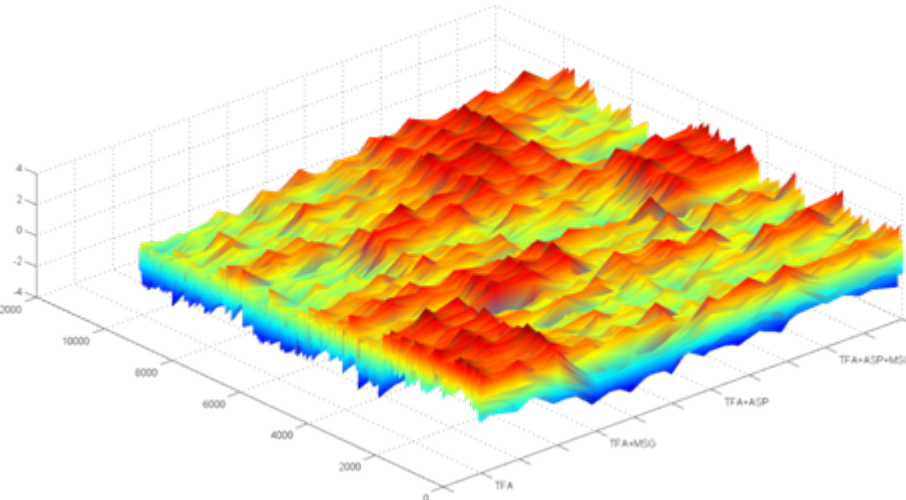

Supplement: Additional file 6 — Diet-specific differences in the functional ontology of genes dysregulated by dietary MSG (A, B); ASP (C, D) and the combination ASP + MSG (E, F). [file 1743-7075-10-44-S6.pdf]
