# Supplementary material for: Prediabetic changes in gene expression induced by aspartame and monosodium glutamate in Trans fat-fed C57Bl/6 J mice
Source: Nutr Metab (Lond). 2013 Jun 19;10:44. doi: 10.1186/1743-7075-10-44 (PMC3727955; doi:10.1186/1743-7075-10-44)
Supplement: Additional file 5 — Diet-specific differences in the number of significant DEGs dysregulated by MSG, ASP, or the combination of ASP + MSG in liver and adipose tissue. [file 1743-7075-10-44-S5.pdf]

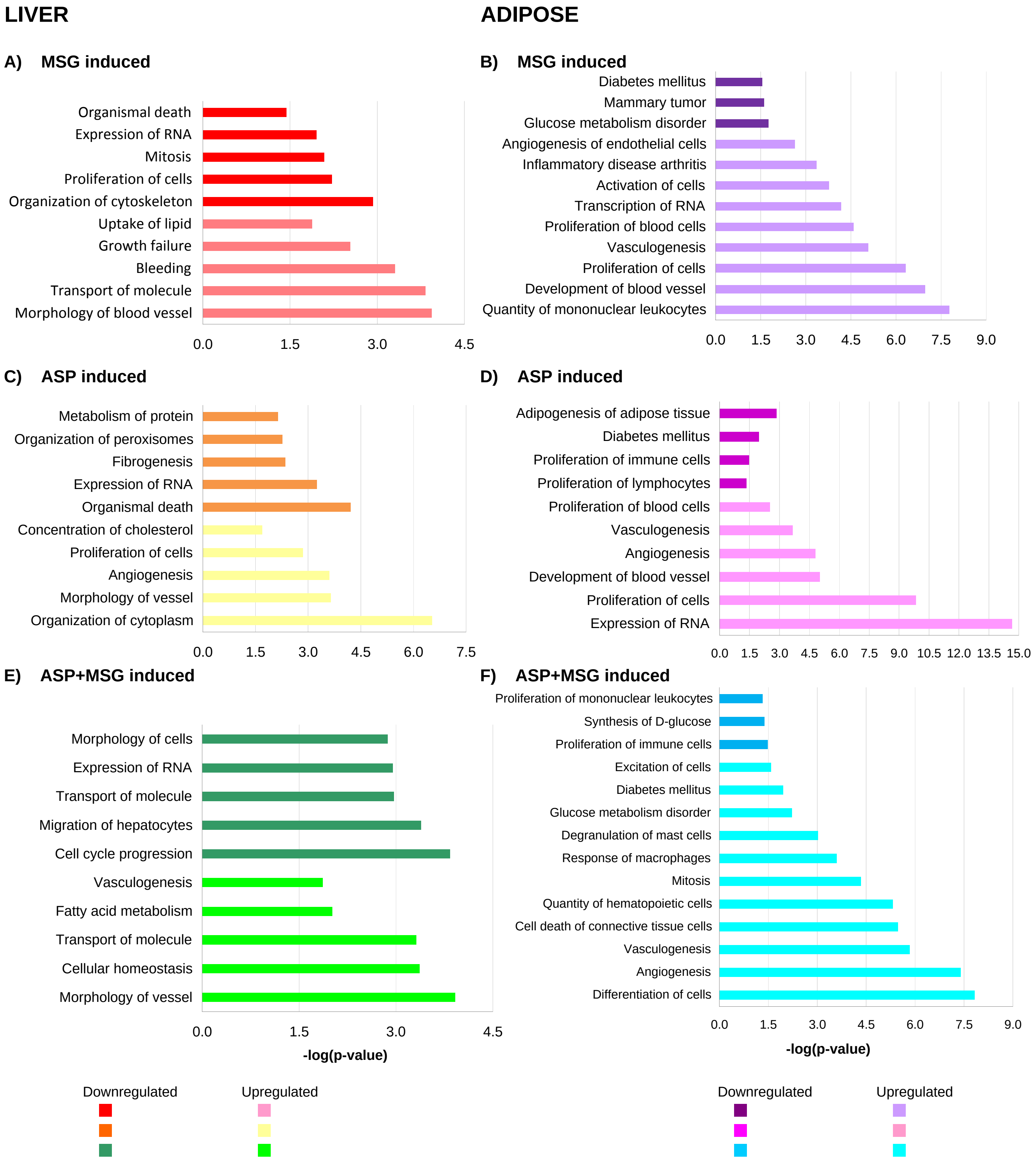
LIVER
ADIPOSE
A) MSG induced
Organismal death
Expression of RNA
Mitosis
Proliferation of cells
Organization of cytoskeleton
Uptake of lipid
Growth failure
Bleeding
Transport of molecule
Morphology of blood vessel
0.0 1.5 3.0 4.5
B) MSG induced
Diabetes mellitus
Mammary tumor
Glucose metabolism disorder
Angiogenesis of endothelial cells
Inflammatory disease arthritis
Activation of cells
Transcription of RNA
Proliferation of blood cells
Vasculogenesis
Proliferation of cells
Development of blood vessel
Quantity of mononuclear leukocytes
0.0 1.5 3.0 4.5 6.0 7.5 9.0
C) ASP induced
Metabolism of protein
Organization of peroxisomes
Fibrogenesis
Expression of RNA
Organismal death
Concentration of cholesterol
Proliferation of cells
Angiogenesis
Morphology of vessel
Organization of cytoplasm
0.0 1.5 3.0 4.5 6.0 7.5
D) ASP induced
Adipogenesis of adipose tissue
Diabetes mellitus
Proliferation of immune cells
Proliferation of lymphocytes
Proliferation of blood cells
Vasculogenesis
Angiogenesis
Development of blood vessel
Proliferation of cells
Expression of RNA
0.0 1.5 3.0 4.5 6.0 7.5 9.0 10.5 12.0 13.5 15.0
E) ASP+MSG induced
Morphology of cells
Expression of RNA
Transport of molecule
Migration of hepatocytes
Cell cycle progression
Vasculogenesis
Fatty acid metabolism
Transport of molecule
Cellular homeostasis
Morphology of vessel
0.0 1.5 3.0 4.5
-log(p-value)
F) ASP+MSG induced
Proliferation of mononuclear leukocytes
Synthesis of D-glucose
Proliferation of immune cells
Excitation of cells
Diabetes mellitus
Glucose metabolism disorder
Degranulation of mast cells
Response of macrophages
Mitosis
Quantity of hematopoietic cells
Cell death of connective tissue cells
Vasculogenesis
Angiogenesis
Differentiation of cells
0.0 1.5 3.0 4.5 6.0 7.5 9.0
-log(p-value)
Downregulated
Upregulated
Downregulated
Upregulated
